# Immunomodulatory potential of mesenchymal stem cell-derived extracellular vesicles: Targeting immune cells

**DOI:** 10.3389/fimmu.2023.1094685

**Published:** 2023-02-13

**Authors:** Xi Liu, Qian Wei, Lu Lu, Shengnan Cui, Kui Ma, Wenhua Zhang, Fang Ma, Haihong Li, Xiaobing Fu, Cuiping Zhang

**Affiliations:** ^1^ Research Center for Tissue Repair and Regeneration Affiliated to the Medical Innovation Research Division and the 4th Medical Center of Chinese PLA General Hospital, Beijing, China; ^2^ Institute of NBC Defence, PLA Army, Beijing, China; ^3^ Department of Dermatology, China Academy of Chinese Medical Science, Xiyuan Hospital, Beijing, China; ^4^ Research Unit of Trauma Care, Tissue Repair and Regeneration, Chinese Academy of Medical Sciences, Beijing, China; ^5^ Department of Ophthalmology, Beijing Chaoyang Hospital, Capital Medical University, Beijing, China; ^6^ Department of Wound Repair, Institute of Wound Repair and Regeneration Medicine, Southern University of Science and Technology Hospital, Southern University of Science and Technology School of Medicine, Shenzhen, China

**Keywords:** extracellular vesicles, mesenchymal stem cells, immune cells, cell dysfunction, inflammatory diseases

## Abstract

Various intractable inflammatory diseases caused by disorders of immune systems have pressed heavily on public health. Innate and adaptive immune cells as well as secreted cytokines and chemokines are commanders to mediate our immune systems. Therefore, restoring normal immunomodulatory responses of immune cells is crucial for the treatment of inflammatory diseases. Mesenchymal stem cell derived extracellular vesicles (MSC-EVs) are nano-sized double-membraned vesicles acting as paracrine effectors of MSCs. MSC-EVs, containing a variety of therapeutic agents, have shown great potential in immune modulation. Herein, we discuss the novel regulatory functions of MSC-EVs from different sources in the activities of innate and adaptive immune cells like macrophages, granulocytes, mast cells, natural killer (NK) cells, dendritic cells (DCs) and lymphocytes. Then, we summarize the latest clinical trials of MSC-EVs in inflammatory diseases. Furthermore, we prospect the research trend of MSC-EVs in the field of immune modulation. Despite the fact that the research on the role of MSC-EVs in regulating immune cells is in infancy, this cell-free therapy based on MSC-EVs still offers a promising solution for the treatment of inflammatory diseases.

## 1 Introduction

Orchestrated responses among variety of innate and adaptive immune cells, organs, cytokines, and chemokines constitute our body’s immune systems to fight against external invasions, together (1). Generally, macrophages, granulocytes, natural killer (NK) cells, mast cells, and dendritic cells (DCs) are called innate immune cells, which can react quickly towards external invasions or injuries. Adaptive immune cells referring to B cells and T cells, are mainly responsible for mediating humoral immunity and cellular immunity, respectively (1). However, the chaotic responses of immune cells often lead to various inflammatory diseases like chronic wounds (2), rheumatoid arthritis (3), inflammatory bowel diseases (4), encephalomyelitis (5), and so on, imposing a heavy burden on the economy and society (6, 7). For example, the unbalanced ratio of anti-inflammatory M2 macrophages (M2φ)/pro-inflammatory M1 macrophages (M1φ) or delayed transition from M1φ to M2φ phenotype of macrophages leads to excessive inflammation, impairing healing process of chronic wounds (8). Therefore, accurately regulating and restoring the behaviors of immune cells are crucial for improving treatment outcomes of inflammatory diseases.

Mesenchymal stem cells (MSCs) are a kind of multipotent stem cells which exist in a wide range of tissues such as bone marrow (BM) (9), adipose tissue (AD) (10), umbilical cord (UC) (11), decidua (12), palatine tonsil (PT) (13), aborted fetal liver (FL) (14), etc. MSCs are believed to exert immunomodulatory or regenerative effects on the injured tissues in various diseases through secreting paracrine factors including extracellular vesicles (EVs) (15). Moreover, as a cell-free bio-entity, MSC-EVs have been recognized as a promising candidate with equal or better therapeutic effect than MSCs.

EVs are nanoscale bodies with cup-like lipid bilayer membranes, containing bioactive components such as lipid, protein, and nucleic acid molecules that are enriched in their parent cells. Based on their origin, size, and bio-genesis, EVs are presently classified into three main categories including exosomes (Exos, size from 50 nm to 100 nm), microvesicles (MVs, 20 nm to 1000 nm), and apoptotic bodies (Abs, 1000 nm to 5000 nm) (16). Proteomics analysis has showed that exclusive markers are expressed highly on their surfaces, such as ALG-2 interacting protein X (Alix), tumor suppressor genes (TSG101), CD9, and CD63 (17). Recently, researchers have investigated the modulation of immune cells by MSC-EVs and explored their clinical potential in the treatment of various inflammatory diseases. Some excellent reviews have discussed the immunomodulatory effects of MSC-EVs on one kind of immune cell like macrophages (18) or on the treatment of inflammatory diseases like liver immunity (19), autoimmune diseases (20), lung diseases (21), rheumatoid arthritis (22) and so on. The accelerated healing process of these inflammatory diseases by MSC-EVs is usually achieved by their immunomodulatory functions on various immune cells. Therefore, it is timely to summarize and discuss the immunomodulatory functions of MSC-EVs on these immune cells systematically.

**Figure 1:**
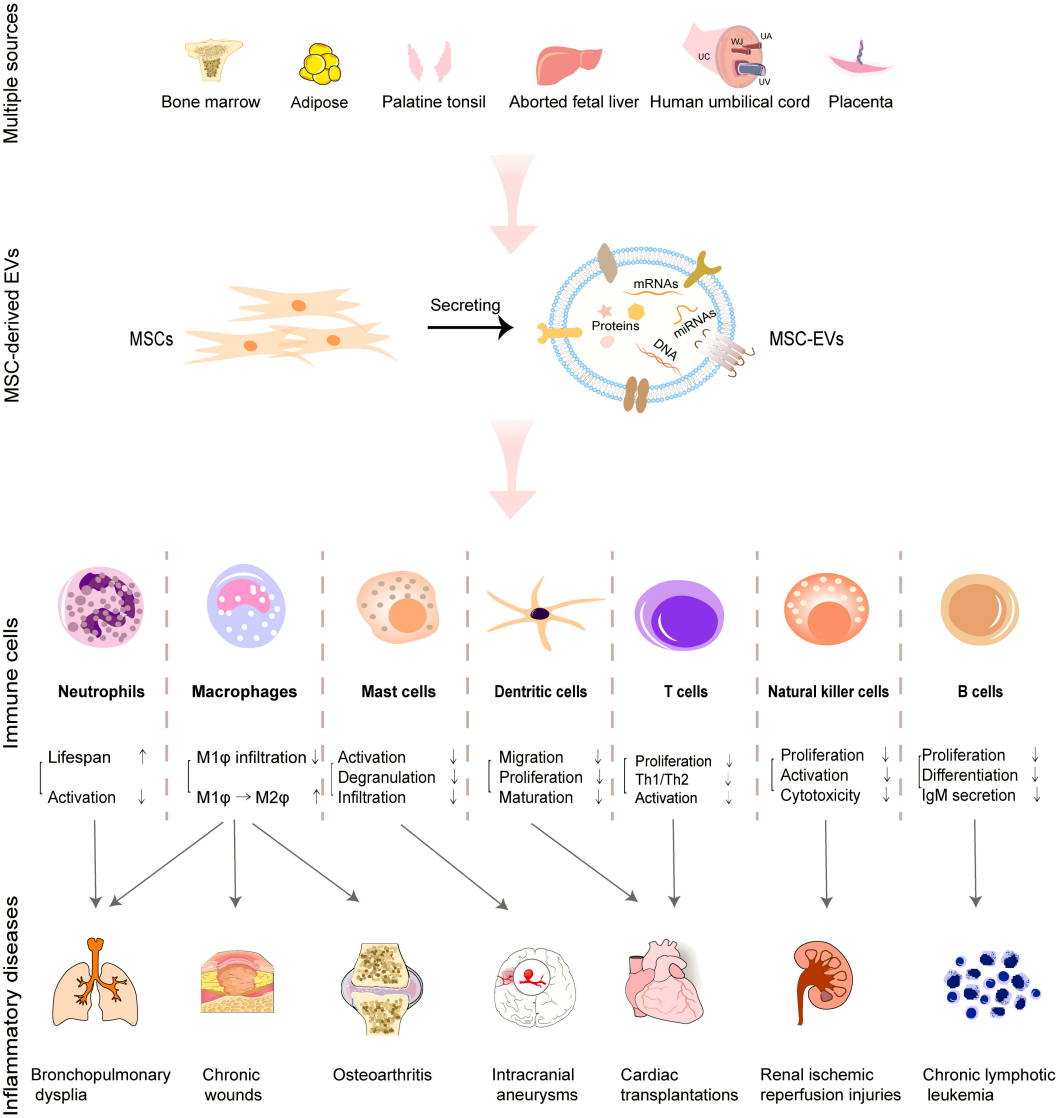
Roles of MSC-EVs derived from multiple tissues in regulating immune cells and facilitating the treatment of associated inflammatory diseases.

In present mini-review, we mainly discuss the immunomodulatory potential and mechanisms of MSC-EVs from multiple sources by targeting innate and adaptive immune cells including macrophages, granulocytes, mast cells, NK cells, DCs and T cells as well as B cells (**Figure 1** and **Table 1**). Additionally, the clinical trials of MSC-EVs administered in different forms for different inflammatory diseases are briefly summarized. Furthermore, the research trends and challenges of MSC-EV applications in inflammatory diseases are presented. Hence, based on the properties and effects on immune cells, MSC-EVs may be developed as a therapeutic strategy for inflammatory diseases.

**Table 1:** Specific pathways and components of MSC-EVs in regulating immune cells.

Targeting cells	Sources of MSCs	Active components	Targeting site/pathways	Key functions	References
Macrophages	hUC	miR-181c	NF-κB	Reduce the number of CD68+ macrophages	(23)
	hUC	has-miR-122-5p, -148a-3p, -486-5p, -let-7a-5p, and 100-5p;	PI3K-AKT signaling pathway	Shift M1φ to M2φ	(24)
	hUC	let-7b	TLR4/NF-κB/STAT3/AKT	Increase macrophage plasticity	(25)
	mBM	miR-let7	miR-let7/IGF2BP1/PTEN and miR-let7/HMGA2/NF-kB	Decrease the infiltration of M1φ and shift M1φ to M2φ	(26)
	hAD	miR-223 and miR-146b	/	Shift M1φ to M2φ	(27)
	cAD	TGF-β and HGF	/	Shift M1φ to M2φ	(28)
Microglia	mBM	/	NF-κB pathway	Promote M2φ polarization and decrease the number of CD68+ macrophages	(29)
	hAD	/	NF-κB and MAPK pathway	Suppress M1φ activation and promote M2φ polarization	(30)
Granulocytes	hWJ	/	/	Enhance the phagocytosis and ROS production	(31)
	hAD	/	/	Recover respiratory burst and prolong the lifespan	(32)
	hESC	/	C5b-9	Downregulate NETs and IL-17	(33)
Mast cells	hAD	/	Serum IgE	Decrease the infiltration of mast cells	(34)
	hBM	/	PGE2 and EP4 receptor	Suppress activation of mast cells	(35)
	hPT	has-miR-214-3p and has-miR-424-5p	/	Suppress activation of mast cells	(13)
	hUC	/	STAT5 phosphorylation	Inhibit the degranulation of mast cells	(36)
NK cells	hUC	/	CX3CL1 and TLR-2	Inhibit the infiltration of NK cells	(37)
	hFL	TSP1	TGF-β/Smad2/3	Inhibit the proliferation, activation, and cytotoxicity of NK cells	(14)
DCs	mBM	/	/	Induce the maturation DCs	(38)
	mBM	/	/	Increase the number of tolerogenic DCs	(39)
	mBM	miR-146a	Fas	Impair the maturation of iDCs and the ability of mDCs to produce IL-12	(40)
	hBM	miR-21-5p	CCR7 gene	Impair the maturation and antigen uptake capacity of iDCs	(41)
	IOD-overexpressing BM	miR-540-3p and FHL-1 protein	JAK3 and AKT	Inhibit the maturation and functions of DCs	(42)
T cells	mBM	PD-L1 and TGF-β	/	Suppress the activation and proliferation of CD4+ T cells	(5)
	cWJ	TGF-β and adenosine	TGF-β and adenosine signaling	Inhibit the mitogen-induced proliferation of CD4+ T cells	(43)
	hEND	TGF-β	TGF-β signaling	Inhibit the activation of CD4+ T cells	(44)
	BM	PGE2 and TGF-β	/	Decrease the number of Th17 cells and increase the number of FoxP3+ Tregs	(45)
	cAD	TSG-6	FOXP3 protein	Increase the number of Tregs	(46)
	mESC	GM-CSF expressing	/	Enhance the migration of CD8+ Teffs and restrict the migration of Tregs	(47)
	hUC/hBM	CD73	adenosinergic signaling	Suppress the proliferation of T cells and promote the apoptosis of CD4+ Th1 cells	(48, 49)
	mAD	β-catenin	Wnt/β-catenin signaling	Promote the migration and circulation of natural killer T cells	(50)
	hBM	miR‐125a‐3p	T cell receptor signaling	Suppress the differentiation of T cells to the effector phenotype	(51)
	IOD-overexpressing BM	miR-540-3p and FHL-1 protein	JAK3 and AKT	Inhibit the maturation and functions of DCs, and regulate T cell immune response in an indirect way	(42)
B cells	hBM	/	IgM	Inhibit the proliferation and differentiation of B cells	(52)
	hBM	/	MZB1 and BCR	Decrease their proliferation of B cells	(53)

hUC, human umbilical cord; mBM, murine bone marrow; hAD, human adipose tissue; cAD, canine adipose tissue; hWJ, human Wharton’s jelly; hESC, human embryonic stem cell; hPT, human palatine tonsil; hFL, human fetal liver; cWJ, canine adipose tissue; hEND, human endometrium; mESC, murine embryonic stem cell, TGF-β, transforming growth factor beta; HGF, hepatocyte growth factor; TSP1, thrombospondin 1; FHL-1, four-and-a-half LIM domain protein 1; PD-L1, programmed death ligand-1; PGE2, prostaglandin E2; TSG-6, tumor necrosis factor-α-stimulated gene/protein 6; GM-CSF, granulocyte-macrophage colonystimulating factor; NF-κB, nuclear factor-κB; PI3K, phosphatidylinositol 3 kinase; PTEN, phosphatase and tensin homolog; MAPK, mitogen-activated protein kinase; C5b-9, terminal complement activation complex; IgE, immunoglobulin E; EP4, E-prostanoid 4; STAT5, signal transducers and activators of transcription5; CX3CL1, C-X3-C motif chemokine ligand-1; TLR-2, toll-like receptor-2; CCR7, C-C chemokine receptor type 7; FOXP3, forkhead box P3; JAK3, Janus kinase 3; IgM, immunoglobulin M; MZB1, marginal zone B1; BCR, B cell receptor; M1φ, M1 macrophages; M2φ, M2 macrophages; ROS, reactive oxygen species; NET, neutrophil extracellular traps; IL-17, interleukin-17; NK cells, natural killer cells, iDCs, immature dendritic cells; mDCs, mature dendritic cells; Tregs, regulatory T cells; Teffs, T effector cells.

Symbol "/" means that the data is not applicable from published references.

## 2 Regulation of immune cell function by MSC-EVs

### 2.1 Innate immune cells

Traditionally, innate immune cells include macrophages, granulocytes, mast cells, NK cells and DCs, which respond quickly to external invasions either by releasing histamine and heparin or by their powerful phagocytosis (1). We mainly focus on the regulation of macrophages, granulocytes, mast cells, NK cells and DCs by MSC-EVs.

#### 2.1.1 Modulation of macrophages or microglia by MSC-EVs

Macrophages exist in almost all tissues of our body and are differentiated from peripheral blood monocytes upon injured tissue recruitment (54). They can not only phagocytose intrusive pathogens but also induce inflammatory response by releasing chemokines (1). Furthermore, they are antigen-presenting cells (APCs) to process and present peptides of phagocytic pathogens on major histocompatibility complex class II (MHC-II) receptor, activating adaptive immune system (54). The macrophages can be reprogramed into two different polarization states to exert their Janus functions, that is classical activated M1φ phenotype (pro-inflammatory) and alternative activated M2φ phenotype (anti-inflammatory) upon the stimulus of cytokines (55). However, the unbalanced M2φ/M1φ ratio or delayed transition from M1φ to M2φ phenotype would lead to continual inflammation (8).

Li et al. observed that EVs derived from human UC-MSCs (hUC-MSC-EVs) reduced the number of macrophages (CD68) to suppress burn-induced inflammation (23). They claimed that miR-181c enriched in hUC-MSC-EVs decreased the expression of toll-like receptor 4 (TLR4), a receptor of lipopolysaccharide (LPS), and subsequently reduced the activation of nuclear factor kappa B (NF-κB)/p65. In another study, hUC-MSC-EVs were also reported to modulate PI3K-AKT signaling pathway to shift macrophage polarization from M1φ to M2φ phenotype by their abundant has-miR-122-5p, -148a-3p, -486-5p, -let-7a-5p, as well as 100-5p (24). Surprisingly, EVs derived from BM-MSCs (BM-MSC-EVs) were reported to achieve “One Stone Two Birds”. They decreased the infiltration number of M1φ phenotype and promoted M2φ phenotype polarization in the plaque of atherosclerosis through miR-let7/IGF2BP1/PTEN and miR-let7/HMGA2/NF-kB signaling pathways, simultaneously (26).

Microglia are a special population of macrophages in the central nervous system. Microglia can not only phagocytose apoptotic neurons but also control synaptic pruning in the brain (56). Microglia can be activated in a phagocytic state under the stimulus of nervous system injuries, releasing lots of cellular inflammatory transmitters (57). Like macrophages, activated microglia can be polarized into pro-inflammatory M1 and anti-inflammatory M2 subtypes, exerting their damage or protection function on neural network (58). Therefore, the proliferation, migration, and activation of microglia are crucial for nerve-related inflammatory responses.

Similarly, BM-MSC-EVs showcased analogical “One Stone Two Birds” modulation on microglia compared with macrophages. BM-MSC-EVs promoted microglial M2 polarization through activating NF-κB pathway and decreased the number of CD68+ microglia (29). In another study, human AD (hAD) derived MSC-EVs were reported to mitigate neuroinflammation mainly by suppressing M1 microglial activation through NF-κB and MAPK pathways and secondarily promoting M2 microglial polarization (30). Moreover, the EVs derived from human retinal progenitor cells were reported to stabilize microglia to avoid its excessive activation, migration, and proliferation, thereby decreasing the secretion of inflammatory cytokines like ionized calcium binding adapter molecule 1 (Iba1) and increasing the anti-inflammatory gene expressions of IL-4, IL-10, and TGF-β, thus mitigating neuroinflammation (59).

In short, the modulation of macrophages or microglia by MSC-EVs is mainly based on two aspects: decreasing their infiltration number and shifting their polarization from M1φ to M2φ subtype, but their potential mechanism still needs to be further explored.

#### 2.1.2 Preconditioning of MSC-EVs

To improve the immunomodulatory functions of MSC-EVs on macrophages, some preconditioning methods, like the stimulus of hypoxia (27, 60), pharmacological agents (25, 61, 62) or pro-inflammatory cytokines (28, 63, 64), have been developed. For example, Sicco et al. pre-exposed hAD-MSCs to hypoxic condition (1% O_2_) and collected the secreted EVs (27). The pretreatment of 1% O_2_ elevated the contents of miR-223 and miR-146b in hAD-MSC-EVs. The hypoxia-changed hAD-MSC-EVs promoted the macrophage polarization from M1φ to M2φ phenotype, and then downregulated the production of interleukin (IL)-6 and Nos2, followed by upregulating the expression of Arg1 and Ym1 *in vivo* and *in vitro*. As for pharmacological agents, Ti et al. claimed that the pretreating of hUC-MSCs with LPS (100 ng/mL) for 48 h enhanced the expression of let-7b inside hUC-MSC-EVs. Let-7b is reported to regulate TLR4/NF-κB/STAT3/Akt pathway which is the potential controller for the macrophage plasticity (25). The precondition of parent cells with pro-inflammatory cytokines was also observed to increase the shifting effect of MSC-EVs on macrophages by improving the contents of the immunoregulatory microRNAs (miRNAs) (63–65) and proteins (28, 66). For example, IL-1β-primed (10 ng/mL, 12 h) hUC-MSCs were observed to secret miR-146a-enriched EVs, which promoted M2φ polarization, efficiently (63). In an interesting study, hUC-MSCs pretreated with tumor necrosis factor alpha (TNFα, 1 ng/mL, 3 d) generated the EVs containing upregulated miRNA-299-3p which accounted for the inhibiting effect of hUC-MSC-EVs on the activation of NLRP3 in macrophages, partially (65). Additionally, preconditioning hAD-MSCs with the mixture of interferon gamma (IFNγ)/TNFα (40 ng/mL, 48 h) upregulated the contents of miR-34a-5p, miR-21 as well as miR-146a-5p inside hAD-MSC-EVs. The obtained hAD-MSC-EVs switched macrophages from M1φ to M2φ phenotype (64). Similarly, in the treatment of experimental murine colitis, preconditioning cAD-MSCs with the mixture of IFNγ/TNFα (20 ng/mL, 24 h) upregulated the expression of immunosuppressive proteins such as hepatocyte growth factor (HGF) and transforming growth factor beta (TGF-β), thus dominating the polarization of macrophages (28).

Collectively, the existing pretreatment methods can efficiently enrich the immunomodulatory components in MSC-EVs. We believe that the exploration to obtain MSC-EVs with more specific components and greater immunomodulation potential *via* milder preconditioning may be the research direction in the future.

#### 2.1.3 Granulocytes

As the main type of phagocytic granulocytes, short-lived neutrophils are recruited to injury sites firstly (1). They phagocytize and destroy pathogens accurately and rapidly by releasing lytic enzymes and producing reactive oxygen species (ROS), further undergoing neutrophil extracellular traps (NETs) (67). Furthermore, the phagocytosis of apoptotic neutrophils can promote anti-inflammatory responses of M2φ subtype and tissue repairing (9).

However, insufficient function and short lifespan of neutrophils often occurred on those patients with severe congenital neutropenia (SCN) (68) or chronic granulomatous disease (CGD) (69), thus leading to serious pathogen infection. Scientists observed the effects of MSC-EVs on the lifespan and biological behaviors of neutrophils from healthy donors, SCN or CGD patients *in vitro* systematically (10, 31, 32, 70). In their studies, the apoptosis and biological behaviors like respiratory burst and phagocytosis of neutrophils were characterized by annexin V-propidium iodide, nitro blue tetrazolium assay, and Giemsa staining. MSCs were isolated from Wharton’s jelly (WJ, a mucosal connective tissue of UC) or from hAD. hAD-MSC-EVs could enhance the phagocytosis and ROS production in neutrophils from both CGD patients and healthy volunteers, and decrease the lifespan of neutrophils from CGD patients (70). However, the influence of hAD-MSC-EVs on neutrophils from SCN patients seemed different. In their observation, hAD-MSC-EVs recovered and prolonged the respiratory burst and lifespan of neutrophils from SCN patients or healthy volunteers significantly, while showed limited influence on the phagocytosis percentage of neutrophils from both SCN patients and healthy volunteers (32). For EVs derived from WJ-MSCs (WJ-MSC-EVs), the lifespan and phagocytosis of neutrophils from healthy volunteers were significantly augmented in comparison with their influence on respiratory burst (31). In-depth studies from aspects of genomics and proteomics should be conducted to explain why those two MSC-EVs showed different regulation behaviors on neutrophils from SCN or CGD patients, or from healthy donors. Very recently, Loh et al. reported that EVs from human embryonic stem cell (hESC, cell line of E1-MYC 16.3)-derived MSCs (hESC-MSC-EVs) inhibited terminal complement activation complex C5b-9-mediated neutrophil activation, thus suppressing the release of NETs and IL-17 *via* a CD59-dependent mechanism (33). This study revealed bright application prospect of hESC-MSC-EVs on treating immune dysregulation in COVID-19 patients.

#### 2.1.4 Other innate immune cells

Mast cells are the first responders of long-lived innate immune cells, that release heparin as well as histamine rapidly in response to an external infection (1). Excessive accumulation and activation of mast cells by immunoglobulin E (IgE) lead to allergy, interstitial cystitis, and other inflammatory diseases (71). Mast cells can be stabilized by MSC-EVs through different molecular mechanisms. Cho et al. observed that treatment with hAD-MSC-EVs (injected either intravenously or subcutaneously) ameliorated the infiltration of mast cells in atopic dermatitis *in vivo* by reducing the level of serum IgE (34). Liu et al. reported that hBM-MSC-EVs suppressed the activation of mast cells (cell line of LAD2) through upregulating the production of prostaglandin E2 (PGE2) and E-prostanoid 4 (EP4) receptors (35). EVs derived from hPT-MSCs (hPT-MSC-EVs) could attenuate TLR7-mediated activation of mast cells. In this study, imiquimod (IMQ, the agonist for TLR7) was employed to activate human mast cell line (HMC-1) (13). The introduction of hPT-MSC-EVs inhibited IMQ-induced HMC-1 activation and the expression of inflammatory cytokines in HMC-1 cells *via* transferring miRNAs like has-miR-214-3p and has-miR-424-5p. Very recently, a study launched by Lin et al. reported that hUC-MSC-EVs suppressed the activation of IgE-stimulated mast cells (cell line of KU812) and downregulated the expression level of NF-κB, thus inhibiting the degranulation of mast cells and release of IL-1β, TNF-α, and IL-6 (36). Furthermore, hUC-MSC-EVs attenuated IgE-induced STAT5 phosphorylation inside KU812 cells in a dose-dependent manner. Collectively, MSC-EVs can stabilize mast cells to relieve allergies by reducing their infiltration and degranulation through different mechanisms.

NK cells are lymphocytes that can detect and kill neighboring infected cells that don’t express a certain number of MHC molecules on cell surface through the specialized receptors on NK cells (NKG2D, KIR, etc.) without prior sensitization (1). MSC-EVs can exert their modulative function on NK cells by regulating their behaviors such as proliferation, activation, and releasing cytotoxic substances. hUC-MSC-EVs were found to relieve the renal ischemic reperfusion injury (IRI) by decreasing the number of NK cells at injury site. hUC-MSC-EVs downregulated the expression of C-X3-C motif chemokine ligand-1 (CX3CL1) and TLR-2, thus inhibiting the infiltration of CD3-CD161+NK cells (37). In another study, hBM-MSC-EVs were also reported to inhibit the secretion of IFN-γ and TNF-α by activating NK cells, showing potential in treating therapy-refractory graft-versus-host diseases (72). EVs derived from human FL (hFL)-MSCs (hFL-MSC-EVs) showed efficient inhibition on the proliferation, activation, and cytotoxicity of NK cells by transferring thrombospondin 1 (TSP1, a regulatory molecule for TGF-β) to downregulate TGF-β/Smad2/3 signaling pathway in NK cells (14). In short, these reports showcased the therapeutic roles of MSC-EVs in inhibiting the lethality of NK cells.

DCs are recognized as the most efficient and professional APCs. They ingest antigens by internalizing invaders and process antigens, followed by presenting antigens to T cells (1). During this process, the antigen-processing immature DCs (iDCs) are transformed to mature DCs (mDCs, antigen-presenting cells) and migrate to secondary lymphoid organs, activating adaptive immune system. Therefore, the immunomodulation on DCs can be achieved by regulating their maturation and migration behaviors. HBM-MSC-EVs induced the hypoactive phenotype of DCs with repressed allorecognition and downregulated expression of costimulatory molecules and MHC-II, subsequently inhibiting the development of Th1 and Th17 cells in the *in-vivo* mouse models of type 1 diabetes and experimental autoimmune uveoretinitis (73). In another study, hBM-MSC-EVs were observed to induce the generation of iDCs, which were characterized by the reduced expression of IL-6 and IL-10 (74). However, the regulated expression of costimulatory markers and MHC-II seemed different in the researches of mAD-MSC-EVs. Cho et al. observed that mAD-MSC-EVs induced the maturation of DCs, characterized by the increased expression of co-stimulatory molecules (38). While, Shahir et al. claimed that the treatment of immature or LPS-induced mature DCs with mAD-MSC-EVs led to the tolerogenic DC population with downregulated expression of costimulatory markers (39). As for underlying molecular mechanisms, several key miRNAs or proteins in MSC-EVs may be involved in the regulation of DC behavior. MBM-MSC-EVs were found to impair the maturation of iDCs and the ability of mDCs to produce IL-12 by transferring miR-146a (the potential miRNA controlling the survival and maturation of human DCs) to iDCs (40). MiR-21-5p was another miRNA that regulated the maturation and function of DCs (41). HBM-MSC-EVs enriched with miR-21-5p degraded the C-C chemokine receptor type 7 gene (CCR7 gene, modulating the homing of DCs to the lymph nodes) and hampered the migration toward the CCR7-ligand CCL21. Furthermore, the treatment with hBM-MSC-EVs restricted the antigen uptake capacity of iDCs and downregulated the secretion of IL-6 and IL-12p70 as well as upregulated the secretion of TGF-β. In another study, the upregulated miR-540-3p and immunoregulatory four-and-a-half LIM domain protein 1 (FHL-1) in EVs derived from indoleamine 2,3-dioxygenase (IDO)-pretreated BM-MSCs were reported to regulate Janus kinase 3 (JAK3, an immune activator) protein negatively and inhibit activation of AKT, respectively, inhibiting the maturation and functions of DCs (42).

### 2.2 Adaptive immune cells

Adaptive immunity usually responds and forms immunological memory by binding with specific pathogens within a few days of disease onset, mainly depending on T or B cells.

#### 2.2.1 T cells

T cells showcase multi-biofunctions including directly killing target cells, regulating antibody production of B cells and secreting lymphokines after the adaptive immunity system is activated (1). Generally, T cells are classified into two subpopulations based on the CD4 and CD8 receptors expressed on their surfaces, called helper and killer T cells respectively. Furthermore, in the presence of IL-12 and IFN-γ, CD4+ T cells are activated into Th1 subtype to induce inflammation and kill pathogens. In the presence of IL-4, CD4+ T cells are activated into Th2 subtype to support the antibody production of B cells (1).

According to literatures, MSC-EVs exerted their immunoregulation functions on T cells with the assistance of APCs. Zhang et al. found that EVs derived from hESC (cell line of huES9.E1)-MSCs (hESC-MSC-EVs) mediated the polarization of Tregs from CD4+ T cells with the assistance of monocytes (75). In their observation, the differentiation of CD4+CD25+FoxP3+ Tregs was observed distinctly by co-incubating hESC-MSC-EVs with CD4+ T cells and human macrophages (THP-1) for 24 h. Furthermore, they found that hESC-MSC-EVs increased the generation of CD4+CD25+Foxp3+ Tregs from CD4+ T cells (activated by APC-enriched spleen cells) through a dose–dependent manner, indicating that MSC-EVs enhanced the production of Tregs through an APC-mediated pathway (76).

A lot of active proteins including tumor necrosis factor-α-stimulated gene/protein 6 (TSG-6) (46), TGF-β (44, 45), adenosine (45, 48), CD73 (48, 49), programmed death ligand-1 (PD-L1) (5), granulocyte-macrophage colony stimulating factor (GM-CSF) (47), and β-catenin (50) loaded in or expressed on the surface of MSC-EVs were involved in the modulation on T cells. As for proteins, TSG-6 in cAD-MSC-EVs was the key protein to increase Tregs by upregulating forkhead box P3 (FOXP3) protein (stabilizes precursor cells of Tregs) (46). TGF-β displayed on the surface of BM-MSC-EVs decreased the number of Th17 cells and increased FoxP3+ Tregs in GAD65-stimulated peripheral blood mononuclear cells (PBMCs) (45). Additionally, TGF-β surface‐bounded or encapsulated in hEND-MSC-EVs exhibited significant inhibition on the activation of CD4+ T cells (44). Mokarizadeh et al. claimed that PD-L1 and TGF-β in mBM-MSC-EVs were responsible for suppressing the activation and proliferation of CD4+ T cells and promoting the generation of Tregs (5). Adenosine and adenosinergic signaling are efficient immunosuppressor and pathway employed by immunosuppressive Tregs by neutralizing pro-inflammatory adenosine 5′-triphosphate (ATP) in the extracellular environment, especially in injured tissues (77). CD73 expressed on the surface of hUC-MSC-EVs (48) or hBM-MSC-EVs (49) catalyzed the production of adenosine from adenosine 5′-monophosphate (AMP), suppressing the proliferation of T cells or promoting the apoptosis of CD39+ Th1 cells. Additionally, Crain et al. claimed that cWJ-MSC-EVs inhibited the mitogen-induced proliferation of CD4+ T cells in a dose-dependent manner with the synergistic effect between TGF-β and adenosine (43). GM-CSF (47) and β-catenin (50) inside MSC-EVs play an important role in immunoregulating T cells associated with tumor therapies. A prophylactic anticancer vaccine composed of GM-CSF-overexpressing mESC-MSC-EVs were reported to enhance the migration of CD8+ T effector cells to rise the expression of pro-inflammatory TNF-α and IFN-γ and restrict the migration of immunosuppressive Tregs (47). Besides, β-catenin inside mAD-MSC-EVs showed significant promotion effect on the migration and circulation of natural killer T cells (50). Furthermore, the stimulus of pro-inflammatory TNF-α and IFN-γ on cAD-MSCs increased the expression of immunosuppressive proteins such as TSG-6, PGE2, and TGF-β inside the derived EVs (28).

Abundant miRNAs inside MSC-EVs were also involved in the regulation process. MiR-125a-3p, which suppresses the proliferation of several cells (78), is the most highly upregulated miRNA inside hBM-MSC-EVs and is recognized to account for the suppression effect of hBM-MSC-EVs on the functional differentiation of T cells (51). MiR-540-3p is another involved miRNA responsible for the regulatory functions of MSC-EVs. He et al. engineered mBM-MSCs by gene transfection to get IDO1-overexpressing mBM-MSC-EVs with upregulated miR-540-3p and FHL-1 protein (42). MiR-540-3p could regulate JAK3, an immune activator, negatively. While, FHL-1 protein was found to suppress IGF/PI3K signaling and activate endoplasmic reticulum (ER) signaling. They cooperated with each other to mediate immunotolerance associated with APCs and T cells after organ transplantation (42).

The roles of T cells and the cooperation between T cells and other immune cells are elaborate and complex. The regulations of the proliferation, migration, activation, apoptosis, and homeostasis of T cells by MSC-EVs should be carefully considered.

#### 2.2.2 B cells

Upon activation and proliferation, B cells can produce large quantities of highly-specific antibodies and secrete them into blood or tissue fluid. Analogously, B cells also include two subtypes, B1 cells (primary producer of natural antibodies, like immunoglobulin M (IgM)) and B2 cells. Furthermore, B2 cells have two subsets including marginal zone B (MZB) cells and follicular B (FOB) cells, which participate in innate and adaptive immune responses, respectively (1).

Controversial results exist about how MSC-EVs regulate B cells. Budoni et al. explored the role of BM-MSC secreted membrane vesicles (MVs, BM-MSC-MVs) in the inhibition of B cells (52). They observed that BM-MSC-MVs specifically inhibited proliferation and differentiation of B cells and suppressed IgM secretion in a dose-dependent manner, compared with T lymphocytes or NK cells. In another study, BM-MSC-EVs were found to decrease the proliferation of B cells *in vitro*, which might be attributed to the upregulated expression level of marginal zone B1 (MZB1) and B cell receptor (BCR)-mediated Ca mobilization in certain subsets of B-lymphocytes (53, 79). Additionally, BM-MSC-EVs promoted migration and chemoresistance of chronic lymphocytic leukemia (CLL) B cells, decreasing their apoptosis in a contact-independent manner by inducing BCR-like activation (80). However, a study launched in 2019 claimed that AD-MSC-EVs isolated by size-exclusion chromatography showed minimal effects on activated B cells, compared with the effects of AD-MSCs, AD-MSC-conditioned medium and AD-MSC derived soluble protein-enriched fractions. AD-MSC-EVs just induced the production of similar CD24^hi^CD38^hi^ B cells, but not real ones because these cells could not produce IL-10 (81). These controversial results may be attributed to different isolation techniques and origins for MSC-EVs in literatures. A standard isolation technology and culturing approach should be established to explore the impacts of MSC-EVs on B cells.

### 2.3 The effect of sources on therapeutic potentials of MSC-EVs

The different sources of MSCs affect the cargoes in EVs like proteins and RNAs (82, 83), thus influencing therapeutic potentials of MSC-EVs (84, 85) in many diseases such as Alzheimer’s disease (86), osteoarthritis (OA) (87), inflammatory response (88), and wound healing (89–91).

However, the comparative studies of MSC-EVs with different origins are relatively limited. HAD-MSC-EVs were reported to cause decreased Aβ peptide level in N2a cells than hBM-MSC-EVs, because hAD-MSC-EVs carried the larger amount of enzymatically active neprilysin (an Aβ-degrading enzyme), showing promising potential in the treatment of Alzheimer’s diseases (86). Zhu et al. compared the treatment efficacy of EVs derived from synovial membrane MSCs (SM-MSC-EVs) with EVs derived from induced pluripotent stem cells (iMSC-EVs) in experimental OA (87). IMSC-EVs showed better therapeutic effects on collagenase-induced OA in mice by promoting the migration and proliferation of chondrocytes than SM-MSC-EVs. The different efficacy was also observed in another study which demonstrated that hBM-MSC-EVs had better therapeutic effects on OA treatment than hAD-MSC-EVs (92). A proteomics analysis showed that differentially expressed proteins (DEPs) between hAD-MSC-EVs and hUC-MSC-EVs were involved in immunity, complement activation, and protein activation cascade regulation in gene ontology (GO) items (93). This is in line with the previous report in which hAD-MSC-EVs, hBM-MSC-EVs, and hUC-MSC-EVs showed prominent immune modulation, regeneration ability, and tissue repair, respectively (94). In aspect of wound healing, the effects of hAD-MSC-EVs and mAD-MSC-EVs were better than that of hBM-MSC-EVs and mBM-MSC-EVs in a diabetic murine model (89, 90). The analysis of cargoes in EVs including proteins and miRNAs explained why hAD-MSC-EVs were closely related to angiogenesis, while hBM-MSC-EVs had more potential to facilitate cellular proliferation. Besides different tissues, age is another influence factor. For example, mBM-MSC-EVs from pre-pubertal group were enriched in miR-21-5p, which was a negative regulator for inflammatory response in macrophages, compared with that of adult groups (88). As for separate studies, the immunomodulation effects of hAD-MSC-EVs, hBM-MSC-EVs, hUC-MSC-EVs on T cells seemed different. HUC-MSC-EVs decreased the migration of CD4+T cells and reduced the percentage of Th1 cells without inducing their apoptosis (95), while BM-MSC-EVs induced the apoptosis of T cells and increased the ratio of Treg/Teff (96). HAD-MSC-EVs were reported to inhibit the proliferation of CD4+ and CD8+ T cells and suppressed the differentiation of CD4+ and CD8+ T cells (97).

In short, in-depth comparative studies are needed to compare the therapeutic potentials of MSC-EVs from different sources. Especially, the culture conditions of MSCs and the isolation methods as well as the administrations and doses of MSC-EVs should be considered carefully in *in-vitro* and *in-vivo* researches to claim which one is more effective than the other.

## 3 Clinical applications of MSC-EVs in inflammatory diseases

Currently, the therapeutic potentials of MSC-EVs have received intense attention in immune therapies, especially during COVID-19 pandemic. According to registered trials on https://www.clinicaltrials.gov, many clinical trials have been launched by different research teams to evaluate the safety and efficacy of MSC-EVs in the treatment of COVID-19 syndrome, SARS-CoV-2 infection, acute respiratory distress syndrome, organ grafting, irritable bowel diseases, burn wounds, osteoarthritis, Alzheimer’s Disease, periodontitis, Type 1 diabetes mellitus and so on (**Table S1**). In a complected study (NCT04493242), one single 15 mL intravenous dose of BM-MSC-EVs increased oxygenation (PaO_2_/FiO_2_) by 192%, reduced about 32% absolute neutrophil count, and increased the number of CD3+, CD4+, and CD8+ lymphocyte by 46%, 45% and 46%, respectively in patients with severe COVID-19 or moderate-to-severe acute respiratory distress syndrome. This result showcased the abilities of MSC-EVs to restore oxygenation, downregulate cytokine storm, and reconstruct immunity system in COVID-19 patients (98). Recently, Shi et al. studied the biodistribution and efficacy of nebulized hAD-MSC-EVs in *Pseudomonas aeruginosa*-induced murine lung injury model and explored the safety of nebulized hAD-MSC-EVs in 24 healthy volunteers (NCT04313647) (99). They found that nebulized hAD-MSC-EVs mitigated lung inflammation and did not cause serious adverse effects on healthy volunteers after the 7th day. These results indicated that MSC-EVs administered in different forms are promising therapeutic candidates in practical treatment of inflammatory diseases.

## 4 Conclusion and perspectives

The regulation of various immune cells by MSC-EVs from multiple sources summarized in this mini-review showcases the great potential of MSC-EVs for the treatment of inflammatory diseases including chronic wounds, osteoarthritis, intestinal diseases, and so on. However, some unrevealed questions still stay in researchers’ minds. The heterogeneity of MSC-EVs is reflective of size, content profile, cellular source, and phenotypic effects on recipient cells. But the exact mechanisms to determine the size, cargo sorting and fate in recipient cells remain elusive. Besides, even for the same group of MSC-EVs, different isolation methods may lead to discrepant net production and purity of MSC-EVs, along with unexpected cell debris or protein deposit, which may bring about deviated effects from genuine impacts mediated by MSC-EVs themselves. Thus, developing a standard and widely-accepted isolation technology of MSC-EVs is very imperative for the scientific community. Furthermore, can we build a research database about the contents in MSC-EVs and their targeting immune cells to realize on-demand design and construction of engineered MSC-EVs by determining the exact components of each MSC-EVs that exert immune regulatory functions? Convincedly, the time has come for MSC-EVs as a novel therapeutic approach for various inflammatory diseases.

## Author contributions

XL, QW, and LL drafted the manuscript. XL and QW prepared the figures. XL, QW, SC, KM, WZ, HL, and FM revised the manuscript. QW and LL revised the figures. XL, HL, XF, and CZ conceptualized, reviewed and funded the manuscript. All authors contributed to the article and approved the submitted version.
