# Supplementary material for: Immunomodulatory potential of mesenchymal stem cell-derived extracellular vesicles: Targeting immune cells
Source: Front Immunol. 2023 Feb 13;14:1094685. doi: 10.3389/fimmu.2023.1094685 (PMC9968735; doi:10.3389/fimmu.2023.1094685)
Supplement: Supplementary file 1 [file Table_1.docx]

**TABLE S1 Recent clinical trials of MSC-EVs in inflammatory diseases.**

| **NCT number** | **Trial purpose** | **Diseases** | **Administration** | **Phase** | **Posted date** | **Status** | **Location** |
| --- | --- | --- | --- | --- | --- | --- | --- |
| 05125562 | Evaluate the safety and efficacy of BM-MSC-EVs | Mild-to-moderate COVID-19 | Intravenous injection | Phase II | November 18, 2021 | Not yet recruiting | United States |
| 05116761 | Evaluate the safety and efficacy of BM-MSC-EVs | Post-acute or chronic post-COVID-19 syndrome | Intravenous injection | Phase I/II | November 11, 2021 | Not yet recruiting | / |
| 04798716 | Explore the safety and efficacy of MSC-EVs | COVID-19 positive patients with acute respiratory distress syndrome and/or novel coronavirus pneumonia | Intravenous injection | Phase I/II | March 15, 2021 | Not yet recruiting | United States |
| 05191381 | Characterize immune  modulatory function of MSC-EVs | Critically ill COVID-19 patients | / | Observational | January 13, 2022 | Recruiting | Germany |
| 05216562 | Evaluate the efficacy and safety of MSC-EVs | Hyper-inflammation in moderate COVID-19 patients | Intravenous injection | Phase II/III | January 31, 2022 | Recruiting | Indonesia |
| 05215288 | Evaluate the safety and improvement in serum inflammatory markers and inflammation of solid abdominal organ by BM-MSC-EVs | Abdominal solid organ  transplant rejection | Intravenous injection | Early phase I | January 31, 2022 | Not yet recruiting | / |
| 05127122 | Evaluate the safety and efficacy of BM-MSC-EVs | Acute respiratory distress syndrome | Intravenous injection | Phase I/II | November 19, 2021 | Not yet recruiting | / |
| 05130983 | Evaluate the feasibility and safety of BM-MSC-EVs | Moderately to severely active Crohn's disease; irritable bowel disease | Intravenous injection | Phase I | November 23, 2021 | Not yet recruiting | / |
| 04493242 | Evaluate the safety and efficacy of BM-MSC-EVs | COVID-19 associated acute respiratory distress syndrome | Intravenous injection | Phase II | July 30, 2020 | Completed | United States |
| 05354141 | Evaluate the safety and efficacy of BM-MSC-EVs | COVID-19 associated moderate-to-severe acute respiratory distress syndrome | Intravenous injection | Phase III | April 29, 2022 | Recruiting | United States |
| 05499156 | Evaluate the safety of human placental MSC-EVs | Resistant perianal fistula in Crohn's patients | Local injection | Phase I/II | August 12, 2022 | Active, not recruiting | Iran |
| 05078385 | Evaluate the safety and efficacy of BM-MSC-EVs | 2nd degree burn wounds | Intravenous injection | Phase I | October 14, 2021 | Not yet recruiting | / |
| 04313647 | Investigate the biodistribution, safety and  eﬀect of nebulized hAD-MSC-EVs | Healthy volunteers | Aerosol | Phase I | March 18, 2020 | Completed | China |
| 04388982 | Evaluate the safety and efficacy of hAD-MSC-EVs | Mild to moderate dementia due to Alzheimer’s disease | Nasal drip | Phase I/II | May 15, 2020 | Recruiting | China |
| 04270006 | Evaluate the regenerative effect of hAD-MSC-EVs | Periodontitis | Local injection | Early Phase I | February 17, 2020 | Recruiting | Egypt |
| 04213248 | Evaluate the effect of hUC-MSC-EVs | Dry eye and chronic graft versus host diseases | Eye drop | Phase I/II | December 30, 2019 | Recruiting | China |
| 04223622 | Evaluate the effects of hAD-MSC secretomes | Osteoarthritis | / | Observational | January 10, 2020 | Recruiting | Italy |
| 02138331 | Evaluate the effect of cell-free cord blood derived EVs | Type 1 diabetes mellitus | / | Phase II/III | May 14, 2014 | Enrolling by invitation | Egypt |
